# Measurement of polarization observables $\textbf{T}$, ${\textbf{P}}$, and ${\textbf{H}}$ in $\mathbf {\pi ^0}$ and $\mathbf {\eta }$ photoproduction off quasi-free nucleons

**DOI:** 10.1140/epja/s10050-023-01134-0

**Published:** 2023-10-17

**Authors:** N. Jermann, B. Krusche, V. Metag, F. Afzal, M. Badea, R. Beck, P. Bielefeldt, J. Bieling, M. Biroth, E. Blanke, N. Borisov, M. Bornstein, K.-T. Brinkmann, S. Ciupka, V. Crede, A. Dolzhikov, P. Drexler, H. Dutz, D. Elsner, A. Fedorov, F. Frommberger, S. Gardner, D. Ghosal, S. Goertz, I. Gorodnov, M. Grüner, C. Hammann, J. Hartmann, W. Hillert, P. Hoffmeister, C. Honisch, T. C. Jude, F. Kalischewski, B. Ketzer, P. Klassen, F. Klein, E. Klempt, J. Knaust, N. Kolanus, J. Kreit, P. Krönert, M. Lang, A. B. Lazarev, K. Livingston, S. Lutterer, P. Mahlberg, C. Meier, W. Meyer, B. Mitlasoczki, J. Müllers, M. Nanova, A. Neganov, K. Nikonov, J. F. Noël, M. Ostrick, J. Ottnad, B. Otto, G. Penman, T. Poller, D. Proft, G. Reicherz, N. Reinartz, L. Richter, S. Runkel, B. Salisbury, A. V. Sarantsev, D. Schaab, C. Schmidt, H. Schmieden, J. Schultes, T. Seifen, K. Spieker, N. Stausberg, M. Steinacher, F. Taubert, A. Thiel, U. Thoma, A. Thomas, M. Urban, G. Urff, Y. Usov, H. van Pee, Y. C. Wang, C. Wendel, U. Wiedner, Y. Wunderlich, CBELSA/TAPS Collaboration

**Affiliations:** 1 Department of Physics, University of Basel, Basel, Switzerland; 2 Helmholtz-Institut für Strahlen-und Kernphysik, University of Bonn, Bonn, Germany; 3 II. Physikalisches Institut, University of Giessen, Giessen, Germany; 4 Institut für Kernphysik, University of Mainz, Mainz, Germany; 5 Joint Institute for Nuclear Research, Dubna, Russia; 6 Department of Physics, Florida State University, Tallahassee, USA; 7 Physikalisches Institut, University of Bonn, Bonn, Germany; 8 SUPA School of Physics and Astronomy, University of Glasgow, Glasgow, UK; 9 Institut für Experimentalphysik I, Ruhr University Bochum, Bochum, Germany; 10 Present Address: resent address: University of Liverpool, Liverpool, UK; 11 Present Address: resent address: University of Hamburg, Hamburg, Germany; 12 Present Address: resent address: Ruhr University Bochum, Bochum, Germany

## Abstract

The target asymmetry *T*, recoil asymmetry *P*, and beam-target double polarization observable *H* were determined in exclusive $\pi ^0$ and $\eta$ photoproduction off quasi-free protons and, for the first time, off quasi-free neutrons. The experiment was performed at the electron stretcher accelerator ELSA in Bonn, Germany, with the Crystal Barrel/TAPS detector setup, using a linearly polarized photon beam and a transversely polarized deuterated butanol target. Effects from the Fermi motion of the nucleons within deuterium were removed by a full kinematic reconstruction of the final state invariant mass. A comparison of the data obtained on the proton and on the neutron provides new insight into the isospin structure of the electromagnetic excitation of the nucleon. Earlier measurements of polarization observables in the $\gamma p \rightarrow \pi ^0 p$ and $\gamma p \rightarrow \eta p$ reactions are confirmed. The data obtained on the neutron are of particular relevance for clarifying the origin of the narrow structure in the $\eta n$ system at $W = 1.68\ \textrm{GeV}$. A comparison with recent partial wave analyses favors the interpretation of this structure as arising from interference of the $S_{11}(1535)$ and $S_{11}(1650)$ resonances within the $S_{11}$-partial wave.

## Introduction

Baryons are composite systems of quarks and gluons. As in any other composite system, the study of the excitation energy spectrum provides information on the interaction among the constituents. Thus, baryon spectroscopy is an important testing ground of quantum chromodynamics (QCD), the non-abelian gauge theory of the strong interaction. While at high momentum transfers ($> 10\ \textrm{GeV}/c$) strong interactions can rather successfully be described by perturbation theory because of the small coupling constant $\alpha _s$, this approach fails at low energies where the coupling constant increases significantly [1]. For the description of baryon excitation energy spectra, QCD-inspired models [2–5] and lattice-QCD calculations [6, 7] have been developed which need to be tested experimentally. These experiments suffer from the complication that the short lifetimes of excited nucleon states lead to widths of the order of $100\ \textrm{MeV}$ [1] and to an overlap of states. Experiments with polarized beams and targets and the interpretation of their results in partial wave analyses (PWAs) have helped disentangle the contributions of different resonances [8].

For a long time, data on the excitation energy spectra of the nucleon were dominated by pion scattering experiments which, however, may have missed resonances that only weakly couple to the $\pi N$ channel. Meanwhile, energy-tagged photon beams at medium-energy electron accelerators have become the predominant experimental tool for investigating resonances in meson production over the last three decades (see [8] for an overview). Not only differential cross sections but also asymmetries measured with polarized photons and polarized targets allow for detailed PWAs like the coupled-channel PWA of Bonn-Gatchina (BnGa) [9, 10], the Scattering Analysis Interactive Dial-in (SAID) [11–13], the unitary Mainz Isobar Model (MAID) [14–16], or the dynamical coupled-channel model of Jülich-Bonn [17]. This provides much more stringent information about the multipoles involved in the reaction, and thus, on the contribution of partially overlapping baryon resonances.

For an unambiguous determination of the isospin decomposition of the amplitudes, photoproduction not only off protons but also off neutrons is crucial. The $\gamma N N^*$ couplings depend on the isospin and differ strongly for protons and neutrons. Here, $\eta$ photoproduction is of special interest for identifying excited states of the nucleon. The isoscalar $\eta$ meson $(I=0)$ ensures only $N^{*} (I=1/2)$ and not $\varDelta (I=3/2)$ contributions as *s*-channel resonances in single $\eta$-photoproduction, which simplifies the data analysis.

A surprisingly narrow structure with a width of $\sim 30\ \textrm{MeV}$ [18] was previously observed around $W = 1.68\ \textrm{GeV}$ in the excitation energy spectrum of the $\eta n$ channel [18–22] but not in the $\eta p$ channel [18, 22, 23]. Different interpretations have been discussed in the literature: an intrinsic resonance [24, 25], interference [26], strangeness loops [27], or coupled channel effects [28, 29]. However, most recent reaction models prefer the interpretation as a resonance [22, 30] or an interference [10, 16]. It has been a prime motivation for the present experiment to provide additional information on the origin of this structure.

Cross section measurements off deuterium and $^3$He report the structure at $W = (1670\pm 5)\ \textrm{MeV}$ with a width of $\varGamma =(30\pm 15)\ \textrm{MeV}$ [18]. A follow-up helicity dependent investigation of the structure [22] showed that it is only observed in the $\sigma _{1/2}$ channel, strongly indicating that only $S_{11}$ and/or $P_{11}$ resonances are involved. A comparison to PWAs of BnGa [26] and the MAID [31] preferred a solution with an additional new narrow $P_{11}$ resonance. A dip in the measured Legendre coefficient $A_{1}$ at the mass of the structure in $\sigma _{1/2}$ (Fig. 3 in [22]) supports this interpretation, whereas the PWA with an interference in the $S_{11}$-wave explaining the narrow structure comparably well agrees with the helicity dependent excitation functions and total cross sections $\sigma _{1/2}$ and $\sigma _{3/2}$ (Fig. 2 in [22]). Thus, it has not yet been clarified which physical phenomenon is responsible for this narrow structure.

In this work, the reactions $\gamma p \rightarrow \pi ^0 p$, $\gamma n \rightarrow \pi ^0 n$, $\gamma p \rightarrow \eta p$, and $\gamma n \rightarrow \eta n$ with the meson decay channels $\pi ^0 \rightarrow 2 \gamma$ (branching ratio $\mathcal{B}\mathcal{R} = (98.82\pm 0.03)\mathrm {\%}$ [1]), $\eta \rightarrow 2 \gamma$ ($\mathcal{B}\mathcal{R} = (39.39\pm 0.18)\mathrm {\%}$ [1]) and $\eta \rightarrow 3 \pi ^0 \rightarrow 6 \gamma$ ($\mathcal{B}\mathcal{R} = (31.43 \pm 0.23)\mathrm {\%}$ [1]) were investigated utilizing a linearly polarized beam and a transversely polarized target. The target nucleons were quasi-free, i.e., bound in the deuterium nuclei of deuterated butanol. The linearly polarized beam photons and transversely polarized target nucleons made the combined determination of the polarization observables *T*, *P*, and *H* possible.

Experiments with polarized neutrons are more complex than measurements with polarized protons. First, free neutrons decay within $\sim 10\ \textrm{min}$ [32, 33], therefore free neutron targets are not available. Second, deuterium, the simplest bound system with a neutron, also contains a proton. To identify the reaction and reconstruct the initial Fermi momentum, the recoil nucleon must be detected, requiring a detector system covering (nearly) the full solid angle. Third, deuterium cannot be polarized easily because it would require very low temperatures ($\sim \ \textrm{mK}$) and high magnetic fields ($\sim 20\ \textrm{T}$) [34] at the same time. A neutron rich and easy-to-handle target material with magnetic-moment-free background nuclei has been chosen here, i.e., deuterated butanol, where moderate magnetic fields of $\sim 2.5\ \textrm{T}$ are sufficient to dynamically polarize the target nuclei. However, this introduces additional dilution from the unpolarized background nuclei that must be controlled.

The paper is structured in the following way: Sect. 2 describes the experimental setup. The various steps in the data analysis are discussed in Sect. 3. The experimental results are presented in Sect. 4 and summarized in Sect. 5, where also the conclusions are given.

## Experimental setup

The data were taken in two beam times at the electron stretcher accelerator ELSA [35] in Bonn, Germany, in 2018 and 2021. The electrons were accelerated to $3.2\ \textrm{GeV}$ before reaching the Crystal Barrel/TAPS experimental site.

Linearly polarized photons were produced at a diamond radiator by the coherent bremsstrahlung process [36] with a coherent edge at $E_{\gamma } = 1200\ \textrm{MeV}$, resulting in a maximum photon polarization degree of $\delta _{\textrm{max}} = 60\mathrm {\%}$ at $1100\ \textrm{MeV}$. Two perpendicular settings of the polarization direction were used, $+45^{\circ }$ (labeled $\parallel$) and $-45^{\circ }$ (labeled $\perp$). The photon energy was inferred from the trajectory of the deflected bremsstrahlung electrons measured in a tagging spectrometer with scintillating fibers and bars in the spectrometer [36].

The detector system consisted of the two main calorimeters Crystal Barrel (CB) composed of 1320 CsI(Tl) crystals [37] covering polar angles from $11^{\circ }$ to $156^{\circ }$ and MiniTAPS (MT) [38] composed of 216 BaF$_2$ crystals from 1$^{\circ }$ to 12$^{\circ }$, together covering $94.6\ \mathrm {\%}$ of the $4\pi$ solid angle. The Inner Detector (ID) [39] comprised 513 plastic scintillator fibers to detect charged particles covering polar angles from $\theta = 14^{\circ }$ to 155$^{\circ }$. For the detection of charged particles covering $11^{\circ }$ to 28$^{\circ }$, 180 plastic scintillators were mounted in front of the most forward 90 CsI(Tl) crystals and 216 plastic scintillators in front of the $\hbox {BaF}_2$ crystals for charged particle detection at $1^{\circ }$ to 12$^{\circ }$. At the end of the beam line, the Gamma Intensity Monitor (GIM) composed of 16 $\hbox {PbF}_2$ crystals and the Flux Monitor (FluMo) monitored the photon flux. FluMo only detected a fraction of the flux and never reached the point of saturation in the experiment, in contrast to the GIM. A $\hbox {CO}_2$ gas Cherenkov detector was positioned between CB and MT, and served as a veto for the trigger signal to suppress electromagnetic background in forward direction. More details of the detector setup are given in [40].

The electronics of CB were upgraded [41] for the 2018 beam time from slow PIN photodiodes to faster avalanche photodiodes and a fast online cluster finder. This made it possible to include CB – for the first time – in the trigger system leading to an increased trigger efficiency for neutral final states. For the 2021 beam time, the charge-to-digital-converters (QDCs) of CB were replaced with FPGA-based sampling analog-to-digital-converters (SADCs) [41], increasing the data taking rate by a factor of two. Without these improvements in the read-out the present experiment would not have been possible.

The Dubna-Mainz Frozen Spin Target [42] was used with deuterated butanol ($\hbox {C}_4$D$_9$OD, named dButanol) as target material, where the nucleons were polarized via the Dynamic Nuclear Polarization (DNP) process [43]. The target was repolarized every few days using a $2.5\ \textrm{T}$ magnet, whereas a $0.6\ \textrm{T}$ holding magnet was used during the data taking. Before and after the repolarization, the degree of target polarization was measured with the nuclear magnetic resonance (NMR) technique [43], and the polarization degree of every run was determined by an interpolation between these two measured values assuming an exponential decrease. A maximum target polarization degree of $\varLambda _{\textrm{max}} = 75\ \mathrm {\%}$ was measured with relaxation times of around $1100\ \textrm{h}$. Two opposite settings of the polarization direction were used, $+90^{\circ }$ (labelled $\uparrow$) and $+270^{\circ }$ (labelled $\downarrow )$. An additional measurement with a carbon foam target was performed with around $20\mathrm {\%}$ of the data of dButanol for the determination of the unpolarized carbon-oxygen dilution. Therefore, only the target material was changed, i.e., the dilution cryostat with the helium was still used to have identical experimental conditions.

Within the trigger, three independent single crystal hit conditions could start the data acquisition: 2 hits in CB, 2 hits in MT, or 1 hit in CB and 1 hit in MT, always with no hit in Cherenkov. Energy thresholds of 16–45 MeV and 80 MeV were set for CB and MT, respectively, depending on the $\theta$ ring for CB.

## Data analysis

The data analysis is done in different steps. First, the raw digital information from analog-to-digital-converters (ADCs) and time-to-digital-converters (TDCs) is converted to (calibrated) physical energy, position and timing information. Second, the coincident detector hits are combined into events. Third, background events are suppressed as much as possible while keeping as many good events as possible by applying kinematic cuts. Fourth, polarization observables are extracted and systematic uncertainties are estimated. This section explains how polarization observables are determined from the measured energy deposits in the detectors. Details on how particle four-momenta are determined and calibrated can be found in [40].

### Particle and reaction reconstruction

#### Event selection

In a presort of the data, particles are reconstructed from the detector hits [40] and combined into possible reactions, applying some conservative kinematic cuts to reduce the computational time for further analysis.

**Table 1:** Required final state multiplicities ($\textrm{c}=\textrm{charged}$, $\textrm{n}=\textrm{neutral}$) in the reconstruction of the different meson decay channels and reactions. The nucleon in bracket is the spectator, which was not detected

Reaction	Meson decay	Multiplicity
$\gamma d\rightarrow \pi ^0 p (n)$	$\pi ^0\rightarrow 2\gamma$	$1\textrm{c}$ and $2\textrm{n}$
$\gamma d\rightarrow \pi ^0 n (p)$	$\pi ^0\rightarrow 2\gamma$	$0\textrm{c}$ and $3\textrm{n}$
$\gamma d\rightarrow \eta p (n)$	$\eta \rightarrow 2\gamma$	$1\textrm{c}$ and $2\textrm{n}$
$\gamma d\rightarrow \eta n (p)$	$\eta \rightarrow 2\gamma$	$0\textrm{c}$ and $3\textrm{n}$
$\gamma d\rightarrow \eta p (n)$	$\eta \rightarrow 3\pi ^0\rightarrow 6\gamma$	$1\textrm{c}$ and $6\textrm{n}$
$\gamma d\rightarrow \eta n (p)$	$\eta \rightarrow 3\pi ^0\rightarrow 6\gamma$	$0\textrm{c}$ and $7\textrm{n}$

For the different reactions different detector hit multiplicities are required as shown in Table 1. Only exclusive reactions are analyzed where both, the decay photons from the meson and the recoil nucleon, must be detected.

The detected neutral particles are combined to create a meson. In $\pi ^0 p$ and $\eta p$ events with $\pi ^0 / \eta \rightarrow 2\gamma$, the invariant mass $m_{\gamma \gamma }$ is calculated assuming the two neutral particles to be photons. Initially, very wide cuts of $50\ \textrm{MeV}< m_{\gamma \gamma } < 250\ \textrm{MeV}$ and $350\ \textrm{MeV}< m_{\gamma \gamma } < 750\ \textrm{MeV}$ are applied to select $\pi ^0$ and $\eta$ mesons, respectively.

In the $\pi ^0 n$ and $\eta n$ channels with $\eta \rightarrow 2\gamma$ the mesons are found by the smallest $\chi ^2$ value of the invariant mass, calculated for all combinations of the 3 neutral hits to construct the meson, defined by

1 $$\begin{aligned} \chi ^2_{ij} = \left( \frac{m_{\gamma _i\gamma _j} - m_{\textrm{PDG}}}{\varDelta m_{\gamma _i\gamma _j}}\right) ^2 \end{aligned}$$

where $m_{\gamma _i\gamma _j}$ is the invariant mass calculated with photon *i* and *j*, $m_{\textrm{PDG}}$ is the nominal mass of the meson taken from [1], and $\varDelta m_{\gamma _i\gamma _j}$ is the uncertainty of the invariant mass, given by the uncertainties of the detected energies and angles. The remaining third neutral hit is the neutron candidate.

The main background in the $\eta n$ channel with $\eta \rightarrow 2\gamma$ are $\pi ^0 n$ events. To suppress them, a $\chi ^2$ anti-cut is applied, i.e., dismissing events where Eq. 1 gives a higher probability for a $\pi ^0$ than an $\eta$ meson for any combination. The same is done for the $\pi ^0 n$ reaction, where $\eta n$ events are suppressed.

In the $\eta \rightarrow 3\pi ^0$ decay channel, the smallest

2 $$\begin{aligned} \chi ^2 = \sum _{i=1}^{3} \left( \frac{m_{\gamma \gamma ,i} - m_{\pi ^0}}{\varDelta m_{\gamma \gamma ,i}}\right) ^2 \end{aligned}$$

is evaluated by calculating all combinations. For $\eta p$ events the charged particle is the proton candidate, for $\eta n$ the remaining $7\textrm{th}$ neutral particle is the neutron candidate.

The energy reconstruction of the meson is optimized with a correction of the decay photons

3 $$\begin{aligned} E^{\prime } = \frac{m_{\textrm{PDG}}}{m_{\gamma \gamma }} E \end{aligned}$$

because of the better angular resolution compared to the energy resolution. For $\eta \rightarrow 3\pi ^0$, this correction is applied for the single $\pi ^0$ as well as for the $\eta$ reconstructed from it.

**Table 2:** Coincidence time cuts between photons ($\gamma -\gamma$), between meson and nucleon ($m-N$), and between tagged electron and meson ($e-m$)

Detector	Type	Cut interval [ns]
CB–CB	$\gamma -\gamma$	$[-40,+40]$
CB –MT	$\gamma -\gamma$	$[-30,+25]$
MT–MT	$\gamma -\gamma$	$[-4,+4]$
CB–CB	$m-N$	$[-50,+60]$
CB–MT	$m-N$	$[-50,+60]$
MT–MT	$m-N$	$[-10,+5]$
Tagger–CB	$e-m$	$[-15, +15]$
Tagger–MT	$e-m$	$[-2, +3]$

**Fig. 1:**
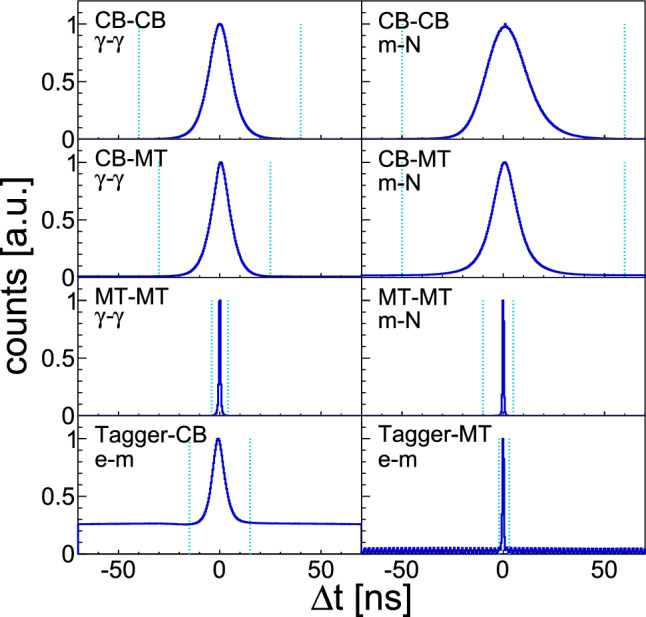
Coincidence times from the reaction $\gamma n \rightarrow \pi ^0 n$. Blue histograms: time difference. Cyan dotted lines: cut positions. Within the canvas, the first text line shows the involved detectors (CB: Crystal Barrel, MT: MiniTAPS, Tagger: Tagging system) and the second one indicates the involved particle ($\gamma$: photon, *m*: meson, *N*: nucleon, *e*: Tagger electron)

The TDC signals from CB allow the application of timing cuts on all events for the first time. As an example, time spectra for the reaction $\gamma n \rightarrow \pi ^0 n$ are shown in Fig. 1. The meson time is defined as the average time of the detected photons – whenever possible selecting MT due to its better time resolution, i.e., if one photon is detected in CB and one in MT the meson time is given by the one of MT only. Wide cuts ($\sim 5\sigma$), listed in Table 2, have been chosen to include all possible good events. Nucleons are slower than photons, which leads to the asymmetry in the cut positions.

The high electron beam current in the experiment of up to $1\ \textrm{nA}$ results in multiple electron hits in the tagging system for one event, which is corrected by a random background subtraction using the side-band method. Every detected electron within the given time window in Table 2 is weighted with a factor of 1, whereas hits in the random background windows of $[-250\ \textrm{ns},\ -50\ \textrm{ns}]$ and $[50\ \textrm{ns},\ 250\ \textrm{ns}]$ are weighted by a factor of $-0.075$ for CB and $-0.0125$ for MT because of the different widths of the prompt signal (Table 2).

#### Background suppression

**Fig. 2:**
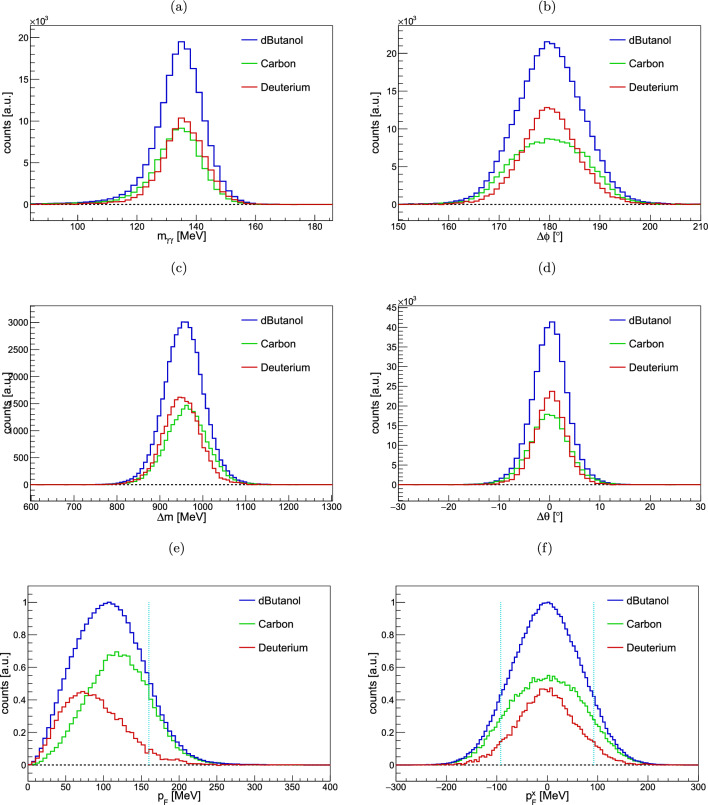
Kinematic background suppression quantities from the reaction $\gamma n \rightarrow \pi ^0 n$. Shown are from top left to bottom right: invariant mass $m_{\gamma \gamma }$, coplanarity $\varDelta \phi$, missing mass $\varDelta m$, polar angle difference $\varDelta \theta$, total Fermi momentum $p_F$, and *x*-component of the Fermi momentum $p_F^x$ for incident photon energies of 650–3100 MeV. All cuts except for the one on the shown quantity are applied. See text for more details. Histograms: deuterated butanol data (blue), carbon data scaled to the photon flux and target densities (green), deuterium data, i.e., difference between deuterated butanol and carbon data (red). Dotted cyan lines: static cut positions for Fermi momenta at $p_F = 160\ \textrm{MeV}$ and $| \vec {p}_F^x | \approx 92.4\ \textrm{MeV}$. See Table 3 for typical cut values

Many different processes may lead to unwanted background events, especially different final states with undetected particles and reactions of unpolarized nuclei. A sophisticated procedure has been developed to remove these events. Energy- and angle-dependent kinematic cuts are performed on the invariant mass, coplanarity, missing mass and polar angle difference. Nuclear Fermi motion is ignored for the calculations of these quantities. The carbon background, however, is scaled by the photon flux (Sect. 3.2) and the difference in the nucleon density in the target (Sect. 3.3), and subtracted from the dButanol data to get the deuterium signal. For getting a very clean signal, all other available cuts are applied for the determination of the individual ones, i.e. a coplanarity, missing mass, polar angle difference and Fermi motion cuts are applied for finding the invariant mass cut. Therefore, the final cut values are found in an iterative procedure. The energy- and angular-dependent kinematic spectra are fit with phenomenological motivated Gaussian-like fit function, as shown in Fig. 2, and $\pm 2.5\ \sigma$ broad cuts are taken. Typical values can be found in Table 3. The angle-dependent mean values and standard deviations are fit with a phenomenological motivated polynomials to achieve continuous cut positions. Furthermore, static Fermi motion cuts are applied. More details can be found in [44]. All kinematic cuts are explained in the following.

**Table 3:** Kinematic background suppression cuts with typical ranges for $\gamma n \rightarrow \pi ^0 n$. The cut positions of the invariant mass, coplanarity, missing mass and polar angle difference are energy- and angle-dependent ($2.5\ \sigma$), and thus, may in some kinematic regions slightly differ from the values given here. See Fig. 2 for the spectra

Kinematic cut quantity	Cut range
Invariant mass $[\textrm{MeV}]$	117.0–153.1
Coplanarity $[{}^{\circ }]$	166.4–193.6
Missing mass $[\textrm{MeV}]$	844.8–1055.7
Polar angle difference $[{}^{\circ }]$	$-7.2$ to 7.2
Total Fermi momentum $[\textrm{MeV}]$	$< 160$
Component Fermi momentum $[\textrm{MeV}]$	$< 92.4$

*Invariant mass:* The invariant mass for $\eta \rightarrow 3\pi ^0$ is calculated with

4 $$\begin{aligned} m_{\eta \rightarrow 3\pi ^0} = \sqrt{\left( \sum _{i=1}^{6} E_{\gamma _i}\right) ^2 - \left( \sum _{i=1}^{6} \vec {p}_{\gamma _i}\right) ^2}, \end{aligned}$$

where $E_{\gamma _i}$ and $\vec {p}_{\gamma _i}$ are the energies and momenta of the decay photons. The fit uses a Gaussian function *G* with an exponential decay towards smaller values, given by [46]

5 $$\begin{aligned}&f\left( x \right) = \left\{ \begin{array}{ll} N G, &{} x\ge \mu \\ N \left( G + \left( 1 - G\right) \exp \left( \frac{x - \mu }{\lambda }\right) \right) , &{} x \le \mu \end{array}\right. \nonumber \\&\textrm{with}\ G = \exp \left( \frac{-(x-\mu )^2}{2\sigma ^2}\right) , \end{aligned}$$

where *N* is a normalization factor, $\mu$ the mean, $\sigma ^2$ the variance and $\lambda$ a decay constant. The angle-dependent mean values are fit with a linear function. The two standard deviations – due to the asymmetric fit function – are fit with a constant function.

*Coplanarity:* The distribution of azimuthal angle difference between the meson and recoil nucleon, called coplanarity, is fit using a Gaussian function with a symmetrical exponential decay in both directions, i.e., the $x\le \mu$ condition in Eq. 5 with $\left( -|x-\mu |/\lambda \right)$ as the argument of the exponential. The angle-dependent mean values are fixed to $180^{\circ }$, where the standard deviations are fit with a quadratic function.

*Missing mass:* Treating the recoil nucleon as a missing particle, its mass can be calculated and should agree with the nominal mass of the recoil nucleon. A Gaussian function is chosen to fit the data. The angle-dependent mean values and standard deviations are both fit with a linear function.

*Polar angle difference*: Again treating the recoil nucleon as a missing particle, its polar angle can be reconstructed from the other particles. The polar angle difference between the measured and reconstructed recoil nucleon is calculated and should be $0^{\circ }$. A Gaussian function is chosen to fit the data. The angle-dependent mean values are fixed to $0^{\circ }$, where the standard deviations are fit with a linear function.

*Fermi momentum*: Additionally, a static Fermi momentum cut is applied on the total and component-wise nucleon momentum. A maximum total Fermi momentum of $p_F = |\vec {p}_F | = 160\ \textrm{MeV}$ is chosen, and a component-wise value of $| \vec {p}_F^{x,y,z} | = | \vec {p}_F | / \sqrt{3}$. The value of $160\ \textrm{MeV}$ is chosen to keep as many good events as possible while dismissing a significant part of carbon and other background events. A smaller value would reduce the number of events drastically, resulting in an increase of the statistical uncertainty, whereas a higher value would include many more background events.

**Fig. 3:**
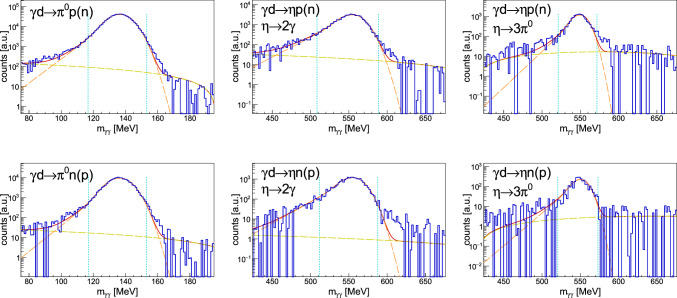
Background contamination determination in the invariant mass $m_{\gamma \gamma }$ spectra for all investigated reactions. Shown are the total angle- and energy-integrated data. Blue histograms: deuterium data. Curves: total fit (solid red) given by Eq. 5 + linear background function for $\gamma d\rightarrow \pi ^0 N (N)$ and $\gamma d\rightarrow \eta N (N)$ with $\eta \rightarrow 2\gamma$, whereas the $\eta \rightarrow 3\pi ^0$ decay channel uses a quadratic background function, fit signal (dashed orange), linear (quadratic) background function (dashed yellow). Dotted cyan lines: $\pm 2.5\sigma$. Note the logarithmic *y*-scale

#### Remaining background contamination

The remaining background contamination is calculated from the invariant mass spectra by assuming an additional linear (or quadratic for $\eta \rightarrow 3\pi ^0$) background function in Eq. 5 resulting in a mean contamination of less than $1\%$ for all reactions except $\eta p$, where the decay channels $\eta \rightarrow 6\gamma$ and $\eta \rightarrow 2\gamma$ have mean background contaminations of $1.4\%$ and $1.2\%$, respectively. Figure 3 shows the good description of the data with the signal and background functions

### Photon flux

The incident-photon flux is given by the electron flux detected in the tagging system times the tagging efficiency, which is the fraction of photons that impinge on the target after passing the collimator. The GIM monitored the photon flux during the entire experiment and delivered the relative photon flux that was scaled to the absolute photon flux values taken once per day with special low-rate runs, where the electron beam current was reduced to reach a GIM rate below $300\ \textrm{kHz}$. These runs were necessary since during the data taking the GIM rate was around $9\ \textrm{MHz}$ resulting in saturation. Furthermore, the GIM efficiency for low-rate runs decreased towards low energies due to the discriminator threshold, which was corrected with the photon flux detected by the FluMo.

The photon flux as a function of the center-of-mass (c.m.) energy *W* is found by convolving the incident-photon flux with the Fermi momentum distribution of nucleons bound in deuterium [47]. For each $E_{\gamma }$-bin one million events are sampled with random isotropic Fermi momentum components satisfying the Fermi momentum distribution of the nucleus and normalized to the number of sampled events.

### Dilution factor

The unpolarized background events coming from reactions off carbon and oxygen nuclei must be taken into account for the extraction of polarization observables. The target polarization degree $\varLambda$ is multiplied by the dilution factor

6 $$\begin{aligned} d = \frac{{\hat{N}}_{\textrm{dB}}-c_t {\hat{N}}_{\textrm{C}}}{{\hat{N}}_{\textrm{dB}}}, \end{aligned}$$

which is given by yields of dButanol (dB) and carbon (C) events normalized to the energy-dependent photon flux (indicated by the hat), $c_t$ is the dilution constant given by the ratio of the different nuclear densities of the frozen-spin target and the carbon foam target.

The scaling factor $c_t$ is determined experimentally because of the large uncertainty in the filling factor along the beam axis. Thereby, coplanarity and missing mass anti-cuts are applied within a Fermi momentum range of 160–$260\ \textrm{MeV}$, where the signal of the carbon is the strongest. The values are $c_t = 0.89\pm 0.01$ and $c_t = 0.85\pm 0.01$ for the two beam times which have relative systematic uncertainties of $12\%$, leading to the systematic uncertainty of the dilution factor [48]

7 $$\begin{aligned} \frac{\varDelta d}{d} = \frac{ 1 - d}{d} \frac{\varDelta c_t}{c_t} \approx 10\mathrm {\%}. \end{aligned}$$

for a mean dilution factor of $\sim 54\%$. The scaling factor agrees with the value calculated from the target densities and is consistent within the uncertainty for all reactions and shows no angle or energy dependency.

### Reconstruction of the final state

The initial Fermi momentum of the target nucleons as well as the kinetic energy of the final state nucleon is not known. Although the energy deposited by the recoil nucleon is measured that value cannot be trusted and has to be reconstructed from the reaction. The deposited energy differs from the true kinetic particle energy in many cases since nucleons are not always fully stopped in the CsI and $\hbox {BaF}_2$ crystals, the crystals are calibrated for photon detection, and because protons also lose a small fraction of energy in the veto detectors and holding structures.

The kinematic reaction can be written as

8 $$\begin{aligned} \gamma + \textrm{D} \rightarrow m + N_P + N_S, \end{aligned}$$

where the photon ($\gamma$) and deuterium nucleus (D) denote the initial state, and the meson (*m*), participant nucleon ($N_P$) and spectator nucleon ($N_S$) in the final state. Ignoring the binding energy of deuterium ($2.2246\ \textrm{MeV}$ [49]), it is assumed within the participant-spectator model that the reaction only occurs on the participant nucleon while the spectator nucleon is not involved in any energy-momentum transfer at all. Thus, the four-momentum of the initial state particles, $p_{\gamma } = \left( E_{\gamma }, 0, 0, E_{\gamma }\right)$ and $p_{\textrm{D}} = \left( m_{\textrm{D}}, 0, 0, 0\right)$, and the known information on the final state meson and participant nucleon (all detected angles, known masses and the energy of the meson) and the known mass of the spectator nucleon fully define the (kinetic) energy of the participant nucleon and the (Fermi) momentum of the initial state nucleons. The information about the meson is reconstructed from the detected photons.

As a result of the energy-momentum conservation of Eq. 8, the kinetic energy of the recoil nucleon is [50, 51]

9 $$\begin{aligned} T_P&= \frac{\sqrt{\left( bc-2a^2m_P \right) ^2 - c^2\left( b^2-a^2\right) }}{2\left( b^2-a^2\right) } \nonumber \\&\quad -\frac{bc-2a^2m_P}{2\left( b^2-a^2\right) } \end{aligned}$$

10 $$\begin{aligned} a&= p_m^x \sin \theta _P\cos \phi _P + p_m^y\sin \theta _P\sin \phi _P \nonumber \\&\quad + \left( p_m^z - E_{\gamma }\right) \cos \theta _P \end{aligned}$$

11 $$\begin{aligned} b&= E_m - E_{\gamma } - m_D \end{aligned}$$

12 $$\begin{aligned} c&= \left( E_m + m_P - E_{\gamma } - m_D \right) ^2 \nonumber \\&\quad - \left( m_S^2 + p_m^2 + E_{\gamma }^2 - 2E_{\gamma }p_m^z \right) , \end{aligned}$$

where $\theta$ and $\phi$ are the polar and azimuthal angles of the recoiling nucleon, which can either be a proton or a neutron. The *true* c.m. energy *W* is then given by the invariant mass of the final state, i.e., $m\left( \pi ^0 N\right)$ and $m\left( \eta N\right)$.

### Extraction of polarization observables

The polarized differential cross section of single pseudoscalar photoproduction with linearly polarized beam photons and transversely polarized target nucleons can be written as [52]

13 $$\begin{aligned} \frac{\textrm{d}\sigma }{\textrm{d}\varOmega }&= \left( \frac{\textrm{d}\sigma }{\textrm{d}\varOmega }\right) _0 \cdot \left\{ 1 -\delta \varSigma \cos \left( 2\left( \alpha -\phi \right) \right) \right. \nonumber \\&\quad + \varLambda T\sin \left( \beta -\phi \right) \nonumber \\&\quad -\delta \varLambda P\cos \left( 2\left( \alpha -\phi \right) \right) \sin \left( \beta -\phi \right) \nonumber \\&\quad - \left. \delta \varLambda H\sin \left( 2\left( \alpha -\phi \right) \right) \cos \left( \beta -\phi \right) \right\} , \end{aligned}$$

in the sign convention of MAID and SAID [53]. Hereby, the photons impinge on the target in the *z*-direction, the 0 index indicates the unpolarized differential cross section, $\alpha$ and $\beta$ are the polarization directions of the linear photon and transverse target polarization in the lab system, $\delta$ and $\varLambda$ are the photon and target polarization degrees, and $\phi$ is the azimuthal angle of the meson in the c.m. system.

The polarization observables are obtained with an event-based unbinned maximum likelihood estimation, i.e., minimizing the negative logarithm of the likelihood function. The probability density function (PDF) is given by Eq. 13 divided by the unpolarized cross section and the $2\pi$ normalization. Additionally to the PDF of interest with the four polarization observables, a PDF for the random background events in the tagger is included, as well as possible detector acceptance asymmetries. The latter are found to be negligibly small. However, they are included anyway. A detailed description of the method used can be found in [10].

For a cross-check, the polarization observables are also extracted with the asymmetry method. The asymmetries, i.e., normalized event yields

14 $$\begin{aligned}{} & {} A_{\varSigma }\left( \phi \right) = \frac{1}{\delta }\frac{{\hat{N}}^{\perp }-{\hat{N}}^{\parallel }}{{\hat{N}}^{\perp }+{\hat{N}}^{\parallel }},\quad A_{T}\left( \phi \right) = \frac{1}{d\varLambda }\frac{{\hat{N}}_{\uparrow }-{\hat{N}}^{\downarrow }}{{\hat{N}}_{\uparrow }+{\hat{N}}_{\downarrow }}, \nonumber \\{} & {} A_{PH}\left( \phi \right) = \frac{1}{d\delta \varLambda }\frac{{\hat{N}}^{\perp }_{\uparrow }-{\hat{N}}^{\perp }_{\downarrow }-{\hat{N}}^{\parallel }_{\uparrow }+{\hat{N}}^{\parallel }_{\downarrow }}{{\hat{N}}^{\perp }_{\uparrow }+{\hat{N}}^{\perp }_{\downarrow }+{\hat{N}}^{\parallel }_{\uparrow }+{\hat{N}}^{\parallel }_{\downarrow }} , \end{aligned}$$

are calculated and fit with the corresponding trigonometrical function from Eq. 13. The beam asymmetry can only be extracted for dButanol and not for nucleons bound in deuterium because they cannot be distinguished from reactions off carbon and oxygen from the dButanol. However, the linear polarization direction $\alpha$ can be extracted, which is used as input parameter in the maximum likelihood fit.

The extracted values from the two beam times are merged and weighted according to their statistical uncertainty.

#### Background correction

The polarization observables are corrected for the background contamination $\delta _{BG}$. The correction and the systematic uncertainty are given as [10]

15 $$\begin{aligned} O = \frac{O_{raw}}{1-\delta _{BG}},\qquad \varDelta O = \frac{1}{\sqrt{3}} \frac{\delta _{BG}}{1-\delta _{BG}}, \end{aligned}$$

where only values with a background contamination of more than $0.5\%$ are corrected, however, a minimum absolute systematic uncertainty of $0.5\%$ is taken for all values. For a completely unpolarized background, Eq. 15 would be exact for *O* and $\varDelta O$ would vanish.

### Systematic uncertainty

The main systematic uncertainty arises from the dilution factor that is around $10\mathrm {\%}$ for the mean dilution factor, where the uncertainty due to the linear polarization is $5\mathrm {\%}$ for $E_{\gamma }=960$–$1310\ \textrm{MeV}$ and $8\mathrm {\%}$ otherwise [54]. The systematic uncertainty of the target polarization is $2.8\%$, which takes into account the NMR measurement and its temperature variation as well as the *D*-wave admixture uncertainty [43]. The background contamination contributes about $1\%$. See Table 4 for an overview of the different sources of systematic uncertainties. The relative uncertainties are converted to absolute ones by convolving the values with a Gaussian function taking the statistical uncertainty into account as standard deviation [10]. In total, the systematic uncertainty results in about $15\%$. Nevertheless, the precision of the data is clearly dominated by the statistical precision except for some kinematic regions of the target asymmetry *T* in the $\pi ^0 p$ channel.

As discussed below (Sect. 4.1) the good agreement in the polarization observables measured on the free proton and on the proton bound in deuterium shows that final state effects are strongly reduced for polarization observables in comparison to absolute cross sections, as already pointed out in [45]. Thus, final state interactions do not seem to significantly enhance the systematic uncertainties. This also implies that the tensor polarization of the deuterium, which could become important in case of strong final state effects, appears to be negligible as for clean quasi-free reactions.

**Table 4:** Sources of systematic uncertainties. The uncertainties of the dilution factor and background contamination are energy- and angle-dependent

Source	Uncertainty $[\%]$
Beam polarization	5–8
Target polarization	2.8
Dilution factor	$\approx 10$
Background contamination	$\approx 1$

## Results

The results are given as a function of $\cos \left( \theta \right)$, where $\theta$ is the polar angle of the meson in the c.m. system, for fixed c.m. energies *W*, which are reconstructed from the final state (Sect. 3.4) and therefore have no Fermi motion smearing. They are compared to the most recent data from other experiments as well as to previously published PWAs of BnGa 2022-02 [10], SAID MA19 [13], and EtaMAID 2018 [16], depending on the reaction. Additionally, BnGa 2022-03 (solid green line), and for $\eta n$ BnGa 2022-03b (dashed green line), are shown, which – in contrast to BnGa 2022-02 (solid black line) and BnGa 2022-02b (dashed black line) – include the results presented here in the fits. The BnGa PWA with the letter b are different from the ones without the letter. They assume the existence of an additional narrow $P_{11}(1680)1/2^+$ state to describe the observed narrow structure in $\eta n$.

**Fig. 4:**
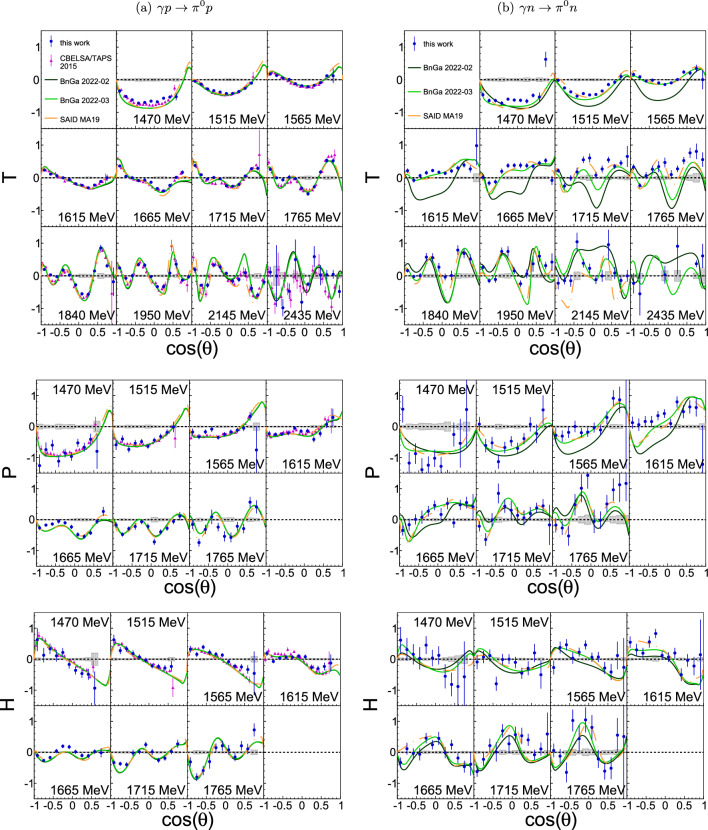
Target asymmetry *T*, recoil asymmetry *P*, and polarization observable *H* as a function of the polar center-of-mass (c.m.) angle $\theta$ of the $\pi ^0$ meson for bins at the given centroid c.m. energies *W*. Left (**a**): $\gamma p \rightarrow \pi ^0 p$. Right (**b**): $\gamma n \rightarrow \pi ^0 n$. Blue circles: this work. Magenta triangles: CBELSA/TAPS data [52]. Gray shaded areas: systematic uncertainties. Curves: model predictions from BnGa 2022-02 (solid black) [10], BnGa 2022-03 (solid green), SAID MA19 (dashed-dotted orange) [13]. BnGa 2022-03 is identical to BnGa 2022-02 but includes the results presented here in the fits

**Fig. 5:**
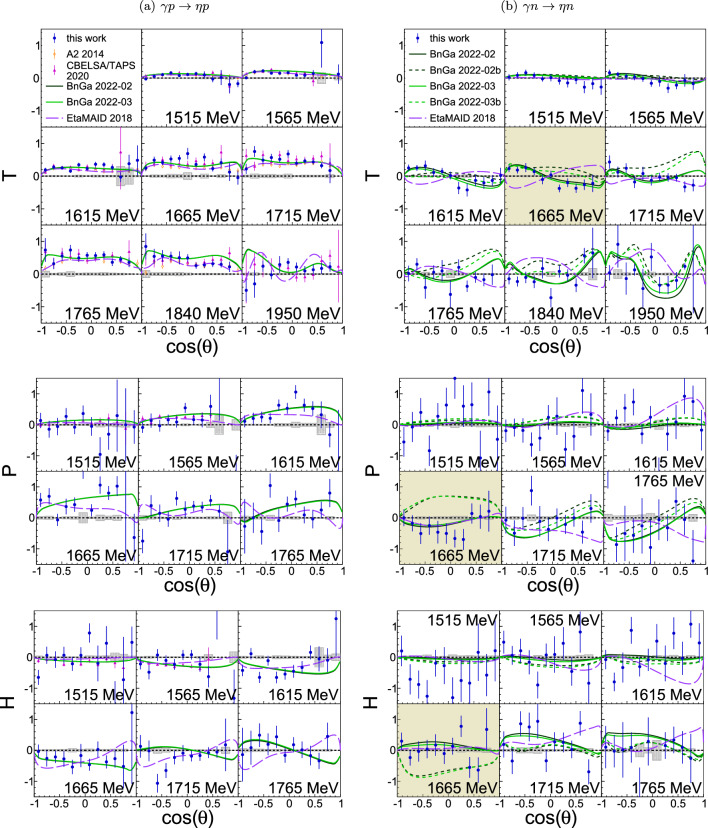
Target asymmetry *T*, recoil asymmetry *P*, and polarization observable *H* as a function of the polar center-of-mass (c.m.) angle $\theta$ of the $\eta$ meson for bins at the given centroid c.m. energies *W*. Left (**a**): $\gamma p \rightarrow \eta p$. Right (**b**): $\gamma n \rightarrow \eta n$. Blue circles: this work. Orange open diamonds: A2 data [56]. Magenta triangles: CBELSA/TAPS data [55]. Gray shaded areas: systematic uncertainties. Curves: PWAs from BnGa 2022-02 (solid black) [10], BnGa 2022-02b (dashed black), BnGa 2022-03 (solid green), BnGa 2022-03b (dashed green curve), EtaMAID 2018 (dashed purple) [16]. The PWAs explain the narrow structure in $\eta n$ around $W=1.68\ \textrm{GeV}$ (yellow bins) as interference of $S_{11}(1535) 1/2^-$ and $S_{11}(1650) 1/2^-$ resonances within the $S_{11}$-partial wave (BnGa 2022-02/BnGa 2022-03), $P_{11}(1680)1/2^+$ resonance (BnGa 2022-02b/BnGa 2022-03b), or $S_{11}(1535) 1/2^- - P_{11}(1710) 1/2^+$ interference (EtaMAID 2018). BnGa 2022-03 is identical to BnGa 2022-02 but includes the results presented here in the fits

**Table 5:** $\chi ^2/\textrm{ndf}$ deviation between the polarization observables and the different partial wave analyses (PWAs) of BnGa, and for $\gamma d\rightarrow \eta n (p)$ of EtaMAID, as shown in Figs. 4 and 5. The total energy ranges for the different reactions and observables can also be seen in Figs. 4 and 5. The phenomenological description of the narrow structure around $W=1.68\ \textrm{GeV}$ in the PWAs from interference of $S_{11}(1535)$ and $S_{11}(1650)$ resonances within the $S_{11}$-partial wave for BnGa 2022-02 and BnGa 2022-03, $P_{11}(1680)$ resonance for BnGa 2022-02b and BnGa 2022-03b, and $S_{11}(1535) - P_{11}(1710)$ interference for EtaMAID 2018. The best values are highlighted

Reaction	PWA	*T*	*P*	*H*	All
*Total energy range*					
$\gamma d\rightarrow \pi ^0 p (n)$	BnGa 2022-02 [10]	6.29	1.85	2.13	3.93
	BnGa 2022-03	$\mathbf {5.77}$	$\mathbf {1.82}$	$\mathbf {1.98}$	$\mathbf {3.64}$
$\gamma d\rightarrow \pi ^0 n (p)$	BnGa 2022-02	26.65	4.26	2.28	13.29
	BnGa 2022-03	$\mathbf {5.20}$	$\mathbf {1.71}$	$\mathbf {1.69}$	$\mathbf {3.20}$
$\gamma d\rightarrow \eta p (n)$	BnGa 2022-02	1.48	1.02	1.28	1.28
	BnGa 2022-03	$\mathbf {1.47}$	$\mathbf {0.99}$	$\mathbf {1.26}$	$\mathbf {1.27}$
$\gamma d\rightarrow \eta n (p)$	BnGa 2022-02	1.57	1.04	$\mathbf {1.12}$	1.28
	BnGa 2022-02b	4.77	1.83	1.51	2.90
	BnGa 2022-03	$\mathbf {1.37}$	$\mathbf {1.03}$	1.13	$\mathbf {1.20}$
	BnGa 2022-03b	2.85	1.73	1.58	2.13
	EtaMAID 2018 [16]	4.73	1.38	1.96	2.63
$W = 1640-1690\ \textrm{MeV}$ ($W = 1590-1740\ \textrm{MeV}$)					
$\gamma d\rightarrow \eta n (p)$	BnGa 2022-02	$1.01\ (1.08)$	$\mathbf {0.74}\ (1.38)$	$1.53\ (1.75)$	$1.10\ (1.39)$
	BnGa 2022-02b	$7.10\ (6.85)$	$8.24\ (3.77)$	$5.51\ (3.14)$	$6.95\ (4.09)$
	BnGa 2022-03	$\mathbf {0.69\ (1.07)}$	$0.84\ \mathbf {(1.37)}$	$1.34\ \mathbf {(1.66)}$	$\mathbf {0.96\ (1.36)}$
	BnGa 2022-03b	$3.83\ (4.55)$	$8.27\ (3.68)$	$6.10\ (3.35)$	$6.07\ (3.63)$
	EtaMAID 2018	$13.32\ (7.63)$	$0.78\ (1.52)$	$\mathbf {1.33}\ (2.68)$	$5.14\ (3.95)$

### Cross-check to previous data in $\gamma p \rightarrow \pi ^0 p$ and $\gamma p \rightarrow \eta p$

All three polarization observables *T*, *P*, and *H* were determined before for the $\pi ^0 p$ and $\eta p$ final states, which therefore become an excellent cross-check for the analysis. The results can be seen in Fig. 4 (a, left column) and 5 (a, left column), where they are compared to previous data from CBELSA/TAPS [52, 55] and for $\eta p$ also to A2 data [56]. BnGa 2022-02 and BnGa 2022-03 are drawn as comparison for both reactions, while SAID MA19 is only available for $\pi ^0 p$ and EtaMAID 2018 only for $\eta p$. Within the uncertainties, all results agree with the existing ones and no larger deviations between the PWAs are visible. It is concluded that the extraction of the polarization observables works fine and the analysis is not biased. Furthermore, final state interactions seem to play only a minor role.

As stated in [55], results reported on *T* in the $\gamma p \rightarrow \eta p$ reaction reported by CBELSA/TAPS [55] are smaller by about a factor of 0.7 compared to the A2 data [56]. The data presented here are in between and agree with both data sets within the uncertainties. However, the difference normalized to the statistical uncertainty slightly prefers the CBELSA/TAPS data, i.e., they are about $6\%$ below the CBELSA/TAPS data but about $16\%$ above the A2 data.

### $\gamma n \rightarrow \pi ^0 n$

For the first time, *T*, *P*, and *H* are determined in $\pi ^0$ photoproduction off neutrons. The results are shown in Fig. 4 (b, right column) and demonstrate the importance of experiments off neutrons. Although BnGa and SAID can both describe the reactions off protons well, they differ here, especially in *T*, where the statistical uncertainty is small. SAID MA19 is in much better agreement to the data than BnGa 2022-02. Including the present results in the BnGa PWA, i.e., BnGa 2022-03 compared to BnGa 2022-02, the agreement improves, resulting in a decrease of the $\chi ^2/\textrm{ndf}$ from 26.65 to 5.20 in *T*, 4.26 to 1.71 in *P* and 2.28 to 1.69 in *H* (Table 5). This improvement is achieved mainly by varying the $\gamma n$ couplings for *P*-waves.

### $\gamma n \rightarrow \eta n$

The BnGa 2022-02b and 2022-03b analyses have been especially performed for the investigation of the previously observed narrow structure in $\eta n$ around $W = 1.68\ \textrm{GeV}$ [18–22] and explains the structure with an additional narrow $P_{11}(1680)1/2^+$ resonance, interfering with the $S_{11}$-wave. The BnGa 2022-02 and BnGa 2022-03 solution describe the narrow structure as arising from interference of $S_{11}(1535) 1/2^-$ and $S_{11}(1650) 1/2^-$ resonances within the $S_{11}$-wave [9, 10], called $S_{11}-S_{11}$ interference in the following, whereas EtaMAID 2018 assumes a $S_{11}(1535) 1/2^- - P_{11}(1710) 1/2^+$ interference [16].

The respective fourth bin in Fig. 5 with the yellow background coloring includes the energy range from $1640\ \textrm{MeV}$ to $1690\ \textrm{MeV}$, and therefore the observed narrow structure. The best agreement to the data is found for BnGa2022-03, i.e., the $S_{11} - S_{11}$ interference, supported by the $\chi ^2/\textrm{ndf}$ values listed in Table 5. The BnGa solution with the additional narrow resonance, BnGa2022-03b, fails to describe all three polarization observables, whereas EtaMAID is in good agreement for *P* and *H* but not for *T*. It should be noted that the improvement achieved in the description of the $\eta$ channel in the BnGa2022-3 solution does not distort but rather improves also the description of the data in the $\pi ^0$ channel.

The $\chi ^2$ values are calculated by combining the statistical and systematical uncertainty quadratically for the data points, i.e., $\sigma _{\textrm{tot}} = \sqrt{\sigma _{\textrm{stat}}^2 + \sigma _{\textrm{syst}}^2}$ for a consistent comparison. The PWAs treat the total uncertainty for their experimental input data in the same way. The reason is that some part of the systematic uncertainty is the same over the whole angular region. A linear combination of the statistical and systematical errors would otherwise lead to an overestimation of the overall uncertainty.

The $\chi ^2/\textrm{ndf}$ deviations to the unpolarized differential cross section [18] are 0.93, 1.87, and 3.5 for BnGa2022-03, BnGa2022-03b and EtaMAID, respectively, over the energy range of $W=1492$–$1875\ \textrm{MeV}$. However, it should be mentioned that the values for the beam asymmetry $\varSigma$ [57] agree better with EtaMAID with $\chi ^2/\textrm{ndf} = 1.8$ (energy range $W=1504$–$1892\ \textrm{MeV}$) compared to 2.22 for BnGa2022-03 (energy range $W=1600$–$1720\ \textrm{MeV}$) and 4.04 for BnGa2022-03b (same energy range as BnGa 2022-03).

## Summary and conclusions

The polarization observables *T*, *P*, and *H* were extracted for $\pi ^0$ and $\eta$ photoproduction off quasi-free protons and, for the first time, off quasi-free neutrons. The good agreement to previous data in the proton data shows that the analysis on quasi-free protons works equally well as for free protons. Experiments with a polarized deuterated butanol target allow for a simultaneous determination of polarization observables off protons and off neutrons, where especially the reactions off polarized neutrons are important for reducing ambiguities in the partial waves.

In the $\eta n$ channel, the comparison to different PWAs strongly prefers an interference of $S_{11}(1535) 1/2^-$ and $S_{11}(1650) 1/2^-$ resonances within the $S_{11}$-partial wave for the description of the narrow structure around $W = 1.68\ \textrm{GeV}$. Only the better agreement of the older BnGa 2014 (b) model with the Legendre coefficient $A_1$ in $\sigma _{1/2}$ [22] would prefer a solution with a narrow $P_{11}$ resonance. However, the other Legendre coefficients and the helicity dependent cross sections are in comparably good agreement with both, BnGa 2014 (b) and the interference interpretation of the narrow structure BnGa 2014 (a). Furthermore, much more data are included in the newer BnGa 2022-02 and BnGa 2022-03 solutions to reduce ambiguities, and therefore give a much better description of the nucleon resonances and their interferences. The interpretation of the narrow structure at $W_{\eta n} = 1.68\ \textrm{GeV}$ as $S-S$ wave interference is therefore preferred and the introduction of a new narrow $P_{11}$ resonance is not needed for a quantitative description of the data.

This measurement clearly shows the importance of (double) polarization measurements and the extracted polarization observables for the identification and correct interpretation of structures in the excitation spectrum of nucleons. In particular, the distinction between interference of already known resonances and possible new resonances can be successfully tested this way.

## Data Availability

This manuscript has associated data in a data repository. [Authors’ comment: The data used in this publication will be available at https://www.hepdata.net].
